# Intermittent theta-burst stimulation to enhance physical therapy in Parkinson's disease: The STEP-PD randomized trial

**DOI:** 10.1016/j.neurot.2026.e00897

**Published:** 2026-04-01

**Authors:** Hong-yu Zhang, Ting-ting Hou, Ke-ke Chen, Tian Zhang, Yi-heng Wang, Yi-xuan Wang, De-tao Meng, Zhen-zhen Li, Hong-jiao Yan, Yi Zhen, Xia An, Rui-dan Wang, Jin-ping Fang, Yong-hong Liu, Wen-jun Du, Jia Du, Ping Wang, Yan-jun Liu, Xiao-yan Yan, Bastiaan R. Bloem, Zhao-hui Jin, Bo-yan Fang

**Affiliations:** a Parkinson Medical Center, Beijing Rehabilitation Hospital, Capital Medical University, Beijing, China; b Capital Medical University, Beijing, China; c Peking University Clinical Research Institute, Peking University First Hospital, Beijing, China; d Radboud University Medical Center, Donders Institute for Brain, Cognition and Behavior, Department of Neurology, Nijmegen, the Netherlands

**Keywords:** Parkinson's disease, Intermittent theta-burst stimulation, Gait, EEG, Randomized trial

## Abstract

Physical therapy (PT) is commonly used to alleviate specific symptoms of Parkinson's disease (PD). Its efficacy may be enhanced by cortical priming, which aims to improve the brain's responsiveness to rehabilitation. This randomized, double-blind, sham-controlled trial—Stimulation to Enhance Physical Therapy in Parkinson's Disease (STEP-PD)—investigated whether combining PT with intermittent theta-burst stimulation (iTBS) applied over the primary motor cortex (M1-iTBS) could provide additional gains in motor function in patients with PD. Fifty participants with PD received PT combined with either bilateral M1-iTBS or sham-iTBS, twice daily, five days per week for two weeks. The primary outcome was the change in Movement Disorder Society-Unified Parkinson's Disease Rating Scale Part III (MDS-UPDRS III) in the OFF-medication state, assessed at baseline and immediately post-intervention. Secondary outcomes included the Parkinson's Disease Questionnaire-39 (PDQ-39), and exploratory outcomes included clinical and instrumented assessments of gait and balance, as well as electroencephalography (EEG)-based measures of functional connectivity. Patients receiving PT combined with M1-iTBS showed greater acute improvement in OFF-state MDS-UPDRS III scores compared to those receiving sham stimulation (Δ = −4.60, *p* = 0.034). There were no significant differences in PDQ-39 scores. Exploratory analyses revealed improved gait stability, fewer falls, and reduced beta-band synchronization on resting-state EEG, suggesting that M1-iTBS may modulate motor networks to facilitate functional recovery. These findings suggest that combining PT with M1-iTBS has promise as an acute cortical priming approach to improve the short-term efficacy of PD rehabilitation, although further research is required to determine the sustainability of these effects.

## Introduction

Parkinson's disease (PD) is a prevalent neurodegenerative disorder marked by progressive dopaminergic neuron loss in the substantia nigra, which leads to tremor, bradykinesia, rigidity, gait disturbances, and postural instability, among other symptoms [1]. These motor impairments substantially diminish quality of life and elevate the risk of falls, fractures, and immobility. Physical therapy (PT) can improve motor function in patients with PD, but access to this treatment remains limited in many parts of the world. Tangible alleviation of motor dysfunction typically requires four weeks of PT or longer [[2], [3], [4]], which further restricts its feasibility for the fast-growing PD population [5].

This study explores the application of ‘cortical priming’ to PD rehabilitation, inspired by the emerging paradigm of neuromodulation-induced cortical prehabilitation (NICP), a strategy originally designed in neuro-oncology to induce functional reorganization before surgical brain insult [[6], [7], [8]]. This priming approach uses transcranial magnetic stimulation (TMS), which induces cortical currents through changing magnetic fields to non-invasively modulate neuronal excitability [9]. Intermittent theta-burst stimulation (iTBS) is a rapid and clinically viable TMS technique associated with prolonged changes in cortical excitability through long-term potentiation (LTP) [10]. In the setting of PD, iTBS targeting the primary motor cortex (M1) may activate M1 and indirectly modulate basal ganglia networks to improve motor control [11]. However, it remains unclear whether such a strategy can effectively enhance rehabilitation outcomes for persons with PD.

Evidence indicates that iTBS can enhance upper limb motor recovery after subacute stroke, particularly when combined with PT, supporting its potential to facilitate neurorehabilitation through cortical priming [12,13]. Whether this strategy can similarly improve outcomes in PD remains unclear. We therefore conducted the randomized, double-blind, sham-controlled clinical trial, Stimulation to Enhance Physical Therapy in Parkinson's Disease (STEP-PD). This study aimed to determine (1) whether bilateral M1-iTBS can enhance the efficacy of PT in improving motor function in patients with PD, and (2) whether this combined intervention modulates neurophysiological markers of cortical excitability and functional connectivity. The study therefore provides preliminary evidence for cortical priming as a strategy to optimize PD rehabilitation.

## Materials and methods

### Experimental design

The STEP-PD study (Chinese Clinical Trial Registry, ChiCTR2200056581, registered in February 2022) was conducted at Beijing Rehabilitation Hospital, Capital Medical University. The trial protocol received approval from the hospital's regulatory and ethics committees. Informed consent was obtained from all participants or their legally authorized representatives. This study adhered to the Consolidated Standards of Reporting Trials (CONSORT 2025) guidelines [14]. Details of the trial design are outlined elsewhere [15].

### Participants

**Fig. 1:**
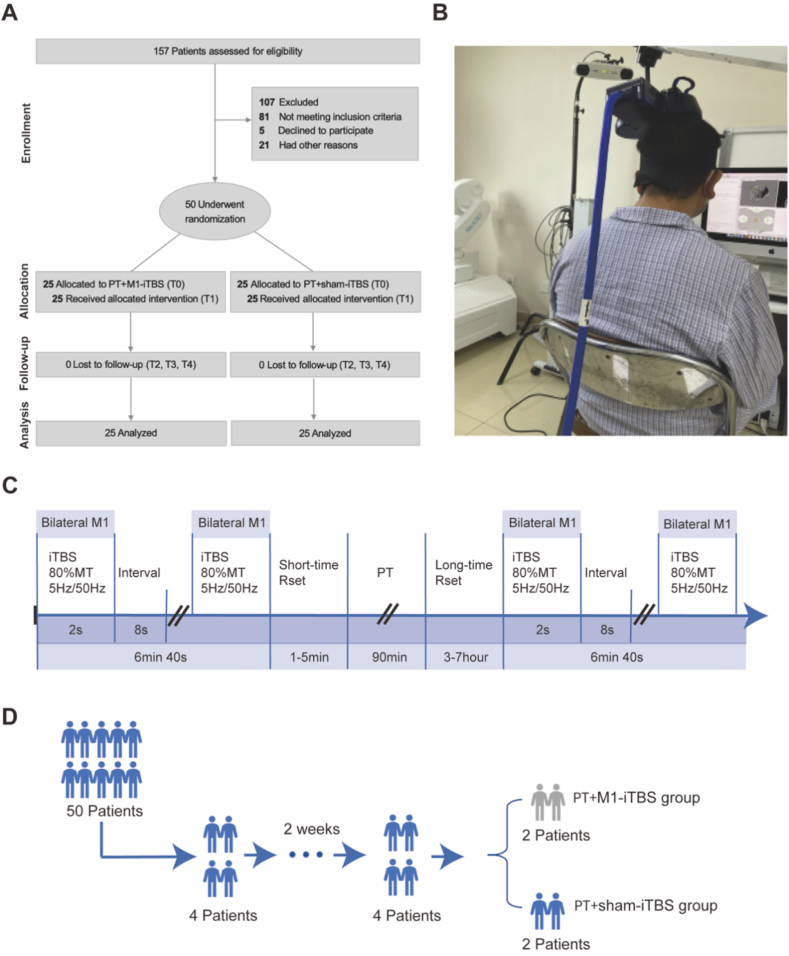
**CONSORT Flow Diagram for Randomization of Patients with Parkinson'sDisease Enrolled in the Study.** (A) CONSORT flowchart denoting details about interventions applied. (B) Patient receiving intermittent theta-burst stimulation. (C) Workflow of iTBS stimulation and PT. (D) Study design.

Between April 14, 2022, and March 22, 2023, 50 patients with PD aged 45–70 years were recruited from Beijing Rehabilitation Hospital. All participants met the 2015 Movement Disorder Society (MDS) diagnostic criteria for PD [16], and were classified as stages 1–3 on the Hoehn and Yahr scale. All were independently ambulatory in daily life but had documented deficits in gait, balance, or functional mobility that made them eligible for referral to PT. All participants were on stable dopaminergic regimens for ≥2 weeks prior to enrollment. Exclusion criteria included other neurological disorders, epilepsy, contraindications to TMS, or having undergone structured PT within the past 6 months. All participants were of Han ethnicity. A participant flow diagram is provided in Fig. 1A.

### Interventions

Both groups underwent a 2-week inpatient treatment protocol comprising PT combined with either M1-iTBS or sham-iTBS, administered 5 days per week. The primary objective was to assess the efficacy and safety of these interventions in facilitating motor recovery in patients with PD.

### Intermittent theta burst stimulation

The iTBS protocol (Magstim Rapid 2, Whitland, UK) followed established guidelines. Stimulation was delivered at 80% of the resting motor threshold (RMT) using 50 Hz triplet pulses at 5 Hz. Each session consisted of 10 cycles of 2-s stimulation with 8-s interstimulus intervals, totaling 600 pulses per hemisphere in 3 min and 20 s. RMT was determined using a figure-eight coil over the M1; the intensity was adjusted until motor-evoked potentials (MEPs) exceeding 50 μV were observed in ≥5 out of 10 trials, or visible thumb contractions were noted [17]. For the sham stimulation condition, a false stimulation coil was used. This coil was designed to simulate sensory experiences associated with real stimulation, such as sound and tactile sensation, without inducing any significant physiological transcranial effects on the brain [18]. It was identical in size, color, and shape to the active coil to ensure blinding. MRI scans were used to localize the M1 target, which was defined on the cortical surface using T1-weighted images and a standardized brain template customized for each participant. Targeting was guided by frameless stereotactic optical navigation (Brainsight, Brainbox Ltd., Cardiff, UK). The coil was positioned tangentially to the skull, oriented in an anterior direction, and precisely centered over the identified M1 target [19]. Participants received two daily sessions, 5 days per week, totaling 20 sessions over 2 weeks, with 3–7 h intervals between sessions (Fig. 1B and C). We based the inter-session interval on previous iTBS studies and conducted a preliminary, non-randomized experiment in a small sample to assess cortical excitability [20,21]. MEPs were recorded at key time points (3, 5, and 7 h) to explore whether excitability changes persisted after two iTBS sessions. These preliminary findings suggested a trend toward enhanced cortical excitability following the two-session iTBS protocol, serving as a pilot reference for the main clinical trial. Detailed methods and MEP amplitude data are provided in Supplement 1 (eTable. 1 and eFig. 1).

### Physical therapy

The PT protocol included four therapeutic interventions, each administered for 30 min daily over 2 consecutive weeks, 5 days per week.

Program 1: Individual PT sessions incorporating warm-up exercises, active and passive joint mobilization to enhance range of motion, abdominal stretching, paraspinal muscle strengthening, postural adjustments, and balance control exercises [22].

Program 2: Daily 30-min balance training using a biofeedback balance device.

Program 3: Gait and balance training using specialized equipment, such as a stability treadmill with biofeedback and virtual reality guidance, gait exercises incorporating virtual obstacles and external cues (*C*-Mill, Motek, Netherlands).

Program 4: Therapist-designed gymnastics specifically tailored for patients with PD, focusing on improving balance, gait, and motor function.

### Outcomes

The primary outcome was change in motor symptoms, evaluated using the Movement Disorder Society-Unified Parkinson's Disease Rating Scale Part III (MDS-UPDRS III) at baseline (T0, within 2 days prior to hospitalization) and immediately post-intervention (T1, within 2 days after treatment completion) [23]. Assessments were conducted in both the OFF-state (primary outcome) and ON-state (secondary outcome). The OFF-state was defined as at least 12 h after the last dose of dopaminergic medication, while the ON-state was assessed approximately 1 h after the patient's usual morning dose.

Secondary outcomes included the 39-item Parkinson's Disease Questionnaire (PDQ-39) for quality of life, assessed at 4 weeks (T2), 12 weeks (T3), and 24 weeks (T4) post-intervention [24]. We also included the following additional exploratory outcomes.

Clinical assessments: 6-Minute Walk Test (6 MWT) to evaluate endurance, a commonly used measure of functional mobility and aerobic capacity in neurodegenerative diseases [25]; and the Gait and Falls Questionnaire (GFQ) to assess fall risk, a tool that evaluates gait abnormalities and fall frequency (score range: 0–64, with higher scores indicating greater gait impairment and fall risk) [26].

Gait Analysis: conducted on an instrumented treadmill (*C*-Mill, Motek, Netherlands) equipped with visual targets and obstacles, assessed during the OFF-medication state. Spatiotemporal gait parameters (step length, width, and stance phase percentage) were measured. Gait tasks comprised six components; each was scored on a scale from 0 to 100, representing the percentage of task completion, with higher scores indicating better performance: visually guided stepping, tandem walking, obstacle avoidance, slalom walking, reaction to perturbation, and speed adaptation. Limits of dynamic stability were assessed by center of pressure area and medial-lateral/anterior-posterior stability. Postural control was evaluated using several tasks (eyes-open, eyes-closed, tandem stance, and single-leg stance), with lower scores indicating better performance. Further details regarding the equipment and methodology are presented in Supplement 2 eFig. 2.

EEG Analysis: EEG data were acquired with a NeuroScan system (NeuroScan, Sterling, VA, USA) with an electrode cap conforming to the international 10–20 system. The signals were referenced to bilateral mastoids, with impedance maintained below 10 kΩ. Data were filtered within a frequency range of 0.05–100 Hz and sampled at a rate of 500 Hz. Ocular and muscle artifacts were identified and removed using independent component analysis combined with visual inspection. Preprocessing involved the extraction of the following frequency bands: theta (4–7 Hz), alpha (8–13 Hz), beta (14–30 Hz), and gamma (31–45 Hz). Power spectral density (PSD) was computed during preprocessing to characterize spectral properties prior to connectivity analysis, but was not treated as an outcome measure. Weighted phase lag index (wPLI) matrices were utilized to evaluate synchronization and connectivity strength, thereby mitigating volume conduction effects and investigating neural responses to iTBS. Both the experimental and control groups underwent EEG assessments to compare cortical activity and connectivity changes before and after the intervention. Additional methodological details are provided in the study protocol.

Safety Evaluation: safety was monitored by recording all adverse events (AEs) during the study.

### Sample size

Sample size calculation was based on previous studies investigating the effects of PT and TMS on MDS-UPDRS III in patients with PD [27,28]. A clinically meaningful improvement for the primary outcome (MDS-UPDRS III) was defined as ≥3.25-point reduction, with the intervention expected to yield a mean reduction of 4.89 points. The control group was assumed to have a mean (SD) score of 4.88 [29,30]. With a 1:1 allocation ratio, the required sample size per group (n) was calculated using the formula: $n=\frac{\left(Z_{\alpha }+Z_{\beta }\right)*2\sigma^{2}}{\delta^{2}}$, where σ is the estimated standard deviation, δ is the expected mean difference, α is the type I error (0.05, two-sided), and β is the type II error (0.10, corresponding to 90% power). This yielded a minimum of 21 participants per group. To account for a 12% dropout rate, 50 participants were enrolled.

### Randomization and blinding

A randomized block design was utilized to ensure the balanced allocation of participants. The 50 participants were stratified into 13 blocks using SPSS 27.0 (IBM, Chicago, IL, USA), with four participants per block, except for the final block, which included two participants. Within each block, participants were randomly assigned to either the PT + iTBS group or the PT + sham-iTBS group (Fig. 1D). To maintain consistency, the same therapist administered all interventions. Clinicians and patients were blinded to group assignments to conform to the rigorous double-blind design. Only the physician responsible for administering iTBS was aware of the group assignments, and had no interaction with participants during stimulation to maintain blinding. All other interventions and assessments were conducted by therapists and physicians who remained blinded to group allocation [15].

### Statistical analysis

Data were analyzed using SPSS Version 27.0. For the primary outcome (MDS-UPDRS III), a two-way mixed-design ANOVA was conducted to examine the interaction between group (PT + M1-iTBS vs. PT + sham-iTBS) and time (T0, T1). The primary analysis focused on between-group differences in score changes, supplemented by within-group comparisons to further characterize treatment effects. Normality assumptions were evaluated using the Shapiro–Wilk test, and data are reported as mean ± standard deviation (mean ± SD). Any violations of sphericity were adjusted using the Greenhouse-Geisser correction, with effect sizes reported as partial eta squared (η^2^p). For secondary outcomes (PDQ-39) and exploratory analyses (GFQ) with non-normal distributions, generalized estimating equations (GEE) were used, with group as the between-subject factor and time (T0, T2, T3, T4) as the within-subject factor. Spatiotemporal gait parameters and stability limits were analyzed using a two-way mixed-design ANOVA after verifying the assumption of normality, reported as mean ± SD. For measures of gait and balance, including the 6 MWT and postural control, which also displayed non-normal distributions, generalized estimating equations were utilized with group and time (T0, T1) as factors. To control the risk of Type I errors arising from the analysis of multiple exploratory gait and balance parameters, *p*-values derived from these analyses were adjusted using the Benjamini–Hochberg false discovery rate (FDR). Adjusted *p*-values are reported, and statistical significance was determined at an adjusted *p* < 0.05. For EEG connectivity analysis, weighted phase lag index matrices were calculated at T0 and T1. Group differences in changes were assessed using independent-sample t-tests. Spearman rank correlation analyses were conducted to examine the relationship between connectivity and behavioral outcomes, with FDR-adjusted *p* < 0.0125 considered significant. For clarity, all *p*-values surviving multiple comparison correction are explicitly labeled as “FDR-adjusted *p*” in the Results and tables, while all other results are reported as nominal *p*-values.

## Results

### Trial population

Between April 14, 2022, and March 22, 2023, 50 patients (median [IQR] age, 65 [59.75–66.25] years; 17 men [34%], 33 women [66%]) were screened and randomized to either the PT + M1-iTBS group or the PT + sham-iTBS group. All participants adhered to their assigned treatments as per the protocol and were included in both the Full Analysis Set and Per-Protocol populations. The trial concluded on October 13, 2023. Baseline characteristics were well-balanced between groups (Table 1). All 25 patients in each group completed their respective treatments and the study (100%). Participants in both groups were on stable PD medication. Eight patients in the real stimulation group and nine in the sham group were taking amantadine. No other *N*-methyl-d-aspartate (NMDA) receptor antagonists or glutamate modulators were used. During follow-up, some patients underwent medication adjustments; however, there was no statistically significant difference in levodopa equivalent daily dose between the groups.

**Table 1:** Baseline characteristics in patients with Parkinson's disease.

Characteristics	Total	Groups	
	(n = 50)	PT + M1-iTBS	PT + sham-iTBS
		(n = 25)	(n = 25)
Demographic			
Age, median (IQR), y	65 (59.75–66.25)	65 (60–66.5)	63 (58.5–67)
Sex, No. (%)			
Male	17 (34)	10 (40)	7 (28)
Female	33 (66)	15 (60)	18 (72)
Hoehn & Yahr stage, No. (%)			
1	1 (2)	1 (4)	0 (0)
2	32 (64)	15 (60)	17 (68)
3	17 (34)	9 (36)	8 (32)
Side of onset, No. (%)			
Left	22 (44)	12 (48)	10 (40)
Right	28 (56)	13 (52)	15 (60)
Time from PD, median (IQR), y	6.5 (5–8.25)	6 (5–8)	7 (5–9)
Education level, median (IQR), y	12 (11–15)	12 (10.5–15)	13 (12–15)
LEDD, median (IQR)	483.25 (395.75–602.00)	466.5 (387.25–576.75)	512.5 (408.00–576.75)
Healthcare expenditure, month median (IQR), CNY	1000 (675–2250)	1000 (1000–3000)	1000 (500–2000)

Abbreviations: M1, primary motor cortex; iTBS, intermittent theta burst stimulation; IQR, interquartile range; PT, physical therapy; PD, Parkinson's disease; LEDD, levodopa equivalent daily dose; CNY, Chinese yuan.

### Primary outcome (MDS-UPDRS III in OFF)

**Fig. 2:**
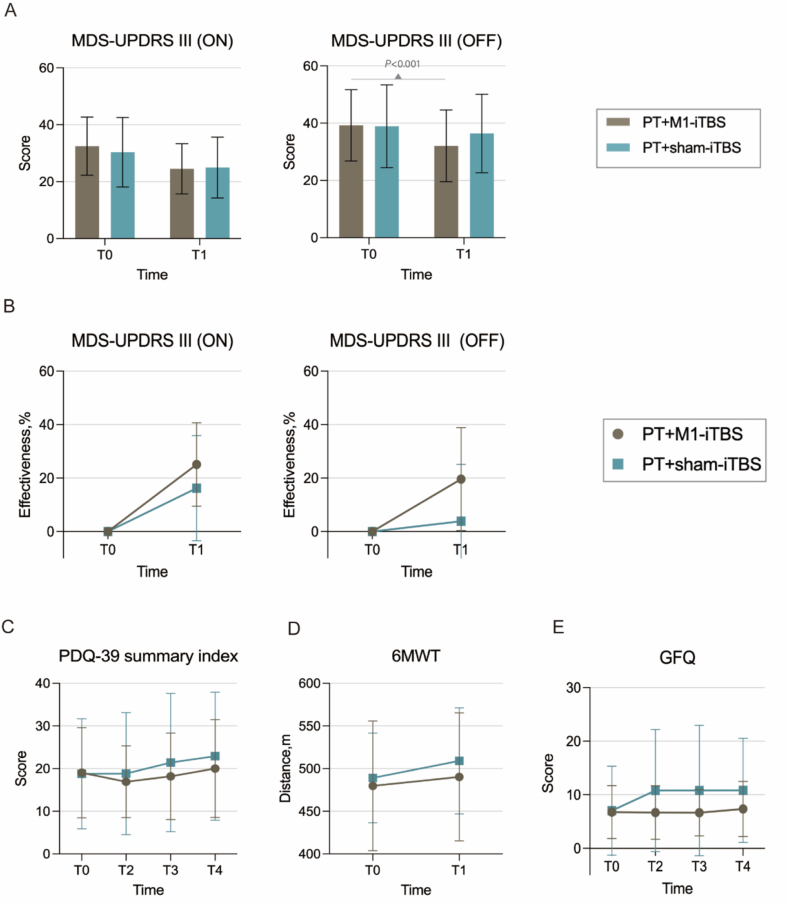
**Effects of M1-iTBS combined with PT on motor and functional outcomes.** (A) MDS-UPDRS III scores in the ON and OFF medication states at baseline (T0) and post-intervention (T1). Significant improvement was observed in the OFF state after PT + M1-iTBS compared with PT + sham-iTBS (P < 0.001). (B) Effectiveness rates (%) of MDS-UPDRS III (ON and OFF) at T0 and T1. (C) PDQ-39 summary index scores across four time points (T0-T4), reflecting health-related quality of life. (D) Six-minute walk test (6 MWT) distances at T0 and T1. (E) Gait and Falls Questionnaire (GFQ) scores across T0-T4. Data are presented as mean ± SD.

A significant time-by-group interaction was observed in the OFF-state (F_1,48_ = 4.785, *p* = 0.034, η^2^p = 0.092, mean difference Δ = −4.60), with a greater improvement in the PT + M1-iTBS group compared to the sham group (Fig. 2A and B). Simple effect analysis revealed that, compared to baseline (T0), PT + M1-iTBS led to a significant reduction in OFF-state MDS-UPDRS III scores at T1 (*p* < 0.001), whereas no significant within-group changes were observed in the sham group (*p* = 0.083) (Supplement 3, eTables 2–3).

### Secondary outcomes

Quality of life was assessed at baseline and 4, 12, and 24 weeks post-treatment. While both PT + M1-iTBS and the PT + sham-iTBS groups exhibited small increases in PDQ-39 scores over time, the changes were not significantly different between the groups (time-by-group interaction, Wald χ^2^ = 2.61, *p* = 0.455) (Fig. 2C).

### Exploratory outcomes

Exploratory outcomes encompassed the 6 MWT, the GFQ, *C*-Mill gait analysis, and EEG analysis. For walking endurance, no significant time-by-group interaction was observed (Wald χ^2^ = 0.455, FDR-adjusted *p* = 0.500) (Fig. 2D). For fall risk, a significant time-by-group interaction was observed for GFQ scores (Wald χ^2^ = 11.396, FDR-adjusted *p* = 0.010), with a greater reduction in fall risk in the PT + M1-iTBS group compared to the sham group (Fig. 2E). Within-group analysis showed that GFQ scores increased significantly at T2, T3, and T4 in the sham group (*p* < 0.01), whereas no significant increases were observed in the PT + M1-iTBS group (Supplement 3, eTable 4).

*C*-Mill gait analysis was conducted at baseline (T0) and post-intervention (T1) to assess gait adaptations. A significant time-by-group interaction was observed for single-stance phase percentage (F_1,48_ = 6.047, FDR-adjusted *p* = 0.018, η^2^p = 0.112) and step length (F_1,48_ = 5.909, FDR-adjusted *p* = 0.019, η^2^p = 0.110), indicating greater improvements in the PT + M1-iTBS group compared to the sham. For single-stance phase percentage, a significant within-group reduction was observed at T1 in the PT + M1-iTBS group (*p* < 0.001). Step length improved in both groups at T1 (PT + M1-iTBS: *p* < 0.001; PT + sham-iTBS: *p* = 0.049), with the PT + M1-iTBS group exhibiting significantly greater improvements than the sham group (*p* = 0.041) (Supplement 3, eTables 5–7).

*C*-Mill stability limits assessment showed that PT + M1-iTBS led to significant improvements in center of pressure area (F_1_, _48_ = 8.828, FDR-adjusted *p* = 0.005, η^2^_p_ = 0.155) and medial-lateral distance (F_1_, _48_ = 7.631, FDR-adjusted *p* = 0.008, η^2^p = 0.137) compared to sham. Simple effects analysis indicated a significant increase in center of pressure area from T0 to T1 in PT + M1-iTBS (*p* < 0.001). At T1, PT + M1-iTBS significantly outperformed sham (*p* < 0.001). Similarly, medial-lateral distance improved significantly in PT + M1-iTBS (*p* < 0.001), with no significant change in sham (*p* = 0.661), resulting in better post-intervention performance for PT + M1-iTBS (*p* < 0.001) (Supplement 3, eTables 5, 8–9).

*C*-Mill postural control analysis revealed that PT + M1-iTBS outperformed sham across eyes-open stance, eyes-closed stance, and single-leg stance tasks (time-by-group interaction: eyes-open stance: Wald χ^2^ = 6.275, FDR-adjusted *p* = 0.012; eyes-closed stance: Wald χ^2^ = 6.065, FDR-adjusted *p* = 0.014; single-leg stance: Wald χ^2^ = 4.277, FDR-adjusted *p* = 0.039). Simple effects analysis showed significant improvements at T1 in PT + M1-iTBS (eyes-open stance: *p* = 0.042; eyes-closed stance: *p* = 0.043; single-leg stance: *p* = 0.035). At T1, PT + M1-iTBS demonstrated better performance than sham (eyes-open stance: *p* < 0.001; eyes-closed stance: *p* < 0.001; single-leg stance: *p* = 0.039) (Supplement 3, eTables 4, 10–12).

*C*-Mill gait task analysis revealed significant time-by-group interactions for visually guided stepping (Wald χ^2^ = 8.167, FDR-adjusted *p* = 0.004) and speed of adaptation (Wald χ^2^ = 6.144, FDR-adjusted *p* = 0.013), with the PT + M1-iTBS group demonstrating superior improvements (visually guided stepping: *p* = 0.038; speed of adaptation: *p* < 0.001). Additionally, a significant group effect was observed for slalom walking, with the PT + M1-iTBS group outperforming sham post-intervention (Wald χ^2^ = 5.921, FDR-adjusted *p* = 0.015) (Supplement 3, eTables 5, 13–14).

### EEG outcomes

**Fig. 3:**
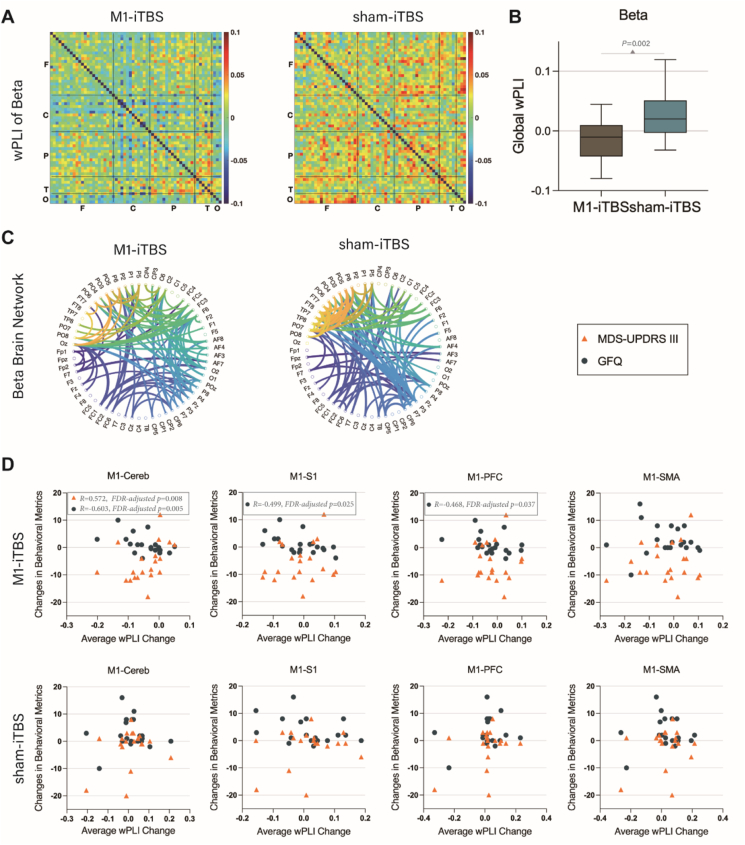
**EEG outcomes.** (A) Functional connectivity difference matrices between T1 and T0 for the PT + M1-iTBS and PT + sham-iTBS groups in the beta frequency band. (B) Group comparison of functional connectivity differences. (C) Circular graphs illustrating the changes in functional connectivity between T1 and T0 in the beta band for both groups. (D) Scatter plots showing the correlations between changes in wPLI functional connectivity (T1-T0) and behavioral metrics (MDS-UPDRS III or GFQ) across different brain regions (M1-Cerebellum, M1-Primary Somatosensory Cortex, M1-Prefrontal Cortex, M1-Supplementary Motor Area) for both groups. Spearman rank correlation was used. Note: The P values displayed in the Figure are nominal values. Statistical significance was determined after FDR correction. For this analysis, FDR-adjusted *P* < 0.0125 was considered significant.

EEG analysis was conducted at baseline (T0) and post-intervention (T1) in both groups. Only 20 patients per group successfully completed the EEG recordings. The weighted phase lag index analysis in the beta frequency band revealed a significant reduction in functional connectivity within the PT + M1-iTBS group compared to the sham group, indicating lower synchronization (*p* = 0.002) (Fig. 3A and B). Additionally, differences in connectivity between specific brain regions were observed (Fig. 3C). Correlational analysis indicated that in the PT + M1-iTBS group, decreased functional connectivity between the M1 region and cerebellum in the beta band was positively correlated with a reduction in MDS-UPDRS III (OFF) scores (R = 0.572, FDR-adjusted *p* = 0.008), and negatively correlated with GFQ scores (R = −0.603, FDR-adjusted *p* = 0.005). No significant correlations were identified in the sham group (FDR-adjusted *p* > 0.0125) (Fig. 3 D).

### Adverse events

iTBS was well-tolerated, with no severe adverse events recorded. One patient in the PT + M1-iTBS group experienced a mild headache, which resolved after rest. No additional adverse events were observed.

### The blinding efficacy

In the PT + M1-iTBS group, 12 participants (48%) believed they had received active stimulation, and 5 (20%) were uncertain. In the PT + sham-iTBS group, 10 participants (40%) judged they had received active stimulation, and 6 (24%) were uncertain. Fisher's exact test showed no significant difference between groups (*p* = 0.78), indicating satisfactory blinding efficacy.

## Discussion

The STEP-PD randomized trial offers preliminary clinical and electrophysiological evidence for cortical priming in PD patients undergoing PT. Our findings indicate that targeted stimulation of the M1 area can modulate acute neural responsiveness in this context. Specifically, when combining PT with M1-iTBS, we observed greater acute between-group improvements in OFF-state MDS-UPDRS III scores at the end of the intervention period. These findings suggest that early cortical modulation may facilitate a more pronounced short-term response during rehabilitation. This provides a potential basis for transitioning from traditional peripheral-focused models toward a central-to-peripheral framework in PD motor therapy.

### Enhanced motor performance through PT combined with M1-iTBS

This study demonstrates that M1-iTBS combined with PT yields an acute improvement of motor function in persons with PD, as evidenced by a greater reduction in MDS-UPDRS III scores in the OFF-state in the intervention group immediately post-intervention. The between-group difference was −4.60 points in favor of active iTBS, exceeding the minimal clinically important difference (3.25 points). This underscores the clinical relevance of the intervention [30]. M1-iTBS did not show significant effects in the ON-medication state, potentially due to a ceiling effect or the masking effect of dopaminergic medication. In the OFF-medication state, the motor network may exhibit heightened sensitivity to cortical excitability modulation, allowing the modulatory effects of iTBS to manifest more prominently [31]. Previous studies have suggested that conventional PT typically requires four or more weeks to produce meaningful motor improvement in PD [2]. In contrast, our two-week protocol achieved clinically significant benefits within a shorter timeframe, suggesting that iTBS may provide an additive benefit that manifests more rapidly than PT alone. However, due to the lack of long-term follow-up data, we cannot determine whether these gains reflect sustained neuroplasticity or an acute physiological summation of the two treatments. While our findings align with prior meta-analytic data on TBS in PD, the durability of these effects beyond the immediate intervention period remains to be established [32].

### Enhancing gait and balance while reducing fall risk

In addition to positive effects on general locomotor skills, gait and postural control also improved significantly. Instrumented gait analysis and clinical balance tasks indicated improvements in spatiotemporal parameters, stability limits, and complex motor adaptability. Concurrent with these gains, GFQ scores exhibited a downward trend during the six-month follow-up period, suggesting a potential reduction in fall-related functional impairment, although these data remain exploratory.

The improvements in gait and balance may result from enhanced cortical responsiveness induced by M1-iTBS, which would theoretically prime neural circuits for more efficient engagement during PT [33]. The intervention enhanced step length and decreased the percentage of single-stance phase on the side initially affected by symptoms—addressing common gait dysfunction in PD [34]—while better performance during stance and dynamic balance tasks points to enhanced postural integration. While both groups exhibited improvements in gait tasks, the PT + M1-iTBS group demonstrated greater benefits in more complex tasks, including visually guided stepping, speed adaptation, and obstacle negotiation. This suggests enhanced adaptability, potentially mediated through targeted modulation of M1 motor circuits [35]. These effects, observed shortly after the two-week protocol, support the role of cortical priming in rapidly enhancing motor readiness prior to task-specific physical training.

### Mechanistic insights: modulation of motor network plasticity

Motor dysfunction in PD is associated with aberrant activity within the cortico-basal ganglia loop. In particular, excessive beta-band synchronization is reported to impair dynamic balance and postural control [36]. Degeneration of nigrostriatal dopaminergic neurons disrupts connectivity between M1 and basal ganglia structures, compromising reflex integration and postural regulation [37].

Our findings are consistent with this pathological role of beta synchronization in motor dysfunction, and suggest that iTBS may reshape cortico-basal ganglia connectivity and attenuate excessive beta-band functional connectivity, restoring motor pathway balance in PD. The observed benefits may reflect an indirect influence of M1-iTBS on basal ganglia circuits, as well as cerebellar and vestibular systems, all of which can contribute to improved symptom control [38]. The modulatory effect of iTBS on brain functioning was supported by the EEG findings, in which a reduction in beta-band functional connectivity (wPLI) correlated with OFF-state MDS-UPDRS III and GFQ scores. We delivered iTBS over M1 to investigate whether it could enhance corticospinal output and thereby optimize response speed and accuracy [39]. The neural effects of iTBS likely involve intricate interactions between M1, the thalamus, and the prefrontal cortex, collectively regulating motor intentions and behavior [40]. iTBS may enhance NMDA receptor-dependent LTP, potentially modulating M1 connectivity and suppressing pathological oscillations [41,42]. Studies in animal models of PD have explored these mechanisms [43]. M1-iTBS may additionally regulate dopamine and glutamate system activity at cortical and subcortical levels, which may also enhance motor network connectivity [42]. In activating dopaminergic pathways and strengthening M1-sensorimotor feedback loop interactions, iTBS has the potential to improve motor control by facilitating more effective postural adjustments, improving gait stability and postural control.

### Cortical excitability modulation does not immediately impact quality of life

We found no improvement in PDQ-39 scores following the intervention. A likely explanation is that motor function gains may require more time to translate into noticeable quality-of-life benefits [44]. Further work with longer follow-up is needed to examine whether sustained motor improvements translate into meaningful functional and psychosocial benefits over time.

### Limitations of the study

This study was not without shortcomings. First, this was a single-center randomized controlled trial, which may limit generalizability. However, the single-center design ensured consistency in treatment delivery, especially important for PT, allowing for more accurate group comparisons. Second, the follow-up period for the primary motor outcome was limited. While we tracked secondary outcomes such as GFQ and PDQ-39 up to 24 weeks, the lack of long-term MDS-UPDRS III assessments is a major limitation. This discrepancy stems from the challenge of conducting in-person, clinician-rated assessments in the OFF-medication state during the COVID-19 pandemic, whereas patient-reported questionnaires could more feasibly be collected remotely. Consequently, we cannot confirm whether the significant acute motor improvements translate into sustained neuroplasticity or represent a transient additive effect. The absence of long-term “gold-standard” motor data necessitates cautious interpretation of the results, as their durability remains unverified. Furthermore, as medication adjustments during follow-up were not systematically quantified at 24 weeks, we acknowledge the potential for residual confounding at the individual level; the long-term outcomes (GFQ/PDQ-39) should therefore also be interpreted with caution. Overall, although the immediate effects are promising, further longitudinal studies are needed to explore the long-term impact and determine whether the observed benefits can be maintained.

Additionally, the absence of a standalone iTBS group limits our ability to distinguish between synergistic interactions and purely additive effects of the two interventions; future factorial designs are needed to isolate these mechanisms. Furthermore, since potential interactions with pharmacological treatments (such as NMDA receptor antagonists) were not explicitly tested, further research is required to clarify how neuromodulation interacts with varied medication regimens.

### Cortical priming as a novel strategy in PD motor rehabilitation

To conclude, this study demonstrates that modulating cortical excitability in conjunction with physical rehabilitation can strengthen short-term motor gains. These findings align with the principles of cortical priming, in which non-invasive stimulation induces the motor network to be more receptive to subsequent PT. The priming approach potentially reinforces the effects of traditional therapy by enhancing sensorimotor integration. Integrating targeted neuromodulation with individualized rehabilitation could thus represent a paradigm shift in PD motor therapy.

While the twice-daily protocol may enhance immediate therapeutic effects, it presents logistical challenges for clinical implementation. The feasibility of our approach was evidenced by high tolerability and a 100% completion rate. However, the reliance on specialized staffing, neuronavigation, and instrumented gait platforms may limit scalability in many settings. Although intensive regimens might reduce hospital stays, this potential cost-benefit remains to be empirically validated. Additionally, future research should examine the optimal timing and sequencing of iTBS relative to PT sessions. Preclinical and human data suggest that neuromodulation preceding task practice can promote cortical receptivity and motor learning, while post-training stimulation could enhance consolidation [45]. Our findings warrant larger trials with extended follow-up to validate cortical priming as a scalable, adjunctive strategy for motor rehabilitation in PD.

## Author contributions

**BYF**: Conceptualization, Methodology, Writing-review & editing, Supervision, Project administration **HYZ**: Writing - original draft, Visualization, Supervision, Data curation, Validation, Formal analysis. **ZHJ**: Methodology, Project administration. **BRB**: Conceptualization, Writing - review & editing. **TTH**: Formal analysis. **XYY**: Formal analysis. **All authors**: Investigation, Data curation, (data collection).

## Data availability

Patient scores underlying the analysis are shared within the manuscript and tables. Due to data privacy regulations concerning patient data, further raw data cannot be publicly shared. Interested parties may request access to the data by contacting the corresponding author (BYF).

## Funding

None.

## Declaration of competing interest

None.
