# Supplementary material for: Intermittent theta-burst stimulation to enhance physical therapy in Parkinson's disease: The STEP-PD randomized trial
Source: Neurotherapeutics. 2026 Apr 1;23(3):e00897. doi: 10.1016/j.neurot.2026.e00897 (PMC13068805; doi:10.1016/j.neurot.2026.e00897)
Supplement: Multimedia component 1 [file mmc1.docx]

Measurement of Motor Evoked Potentials (MEP)

To assess cortical excitability changes during the inter-session interval of two daily iTBS treatments, a preliminary experiment was conducted with 8 subjects (n=8). Despite the small sample size, the data show that cortical excitability remained elevated throughout the inter-treatment period, supporting the effectiveness of the chosen treatment interval.

TMS Procedure and Settings

Single-pulse TMS was delivered using a Magstim Rapid2 system (Magstim, UK) with a figure-of-eight coil, with the coil positioned posteriorly. The stimulation site was the primary motor cortex, corresponding to the contralateral first dorsal interosseous (FDI) muscle. Stimulation intensity was set relative to the resting motor threshold (RMT) for each participant. Surface electromyography (EMG) signals were recorded using a Digitimer D360 amplifier (Digitimer, UK) and digitized using a CED 1401 system (Cambridge Electronic Design, UK), then stored for offline analysis. RMT and active motor threshold (AMT) were measured for each participant, and the stimulation intensity was set to evoke MEPs with ~1 mV amplitude.

At each time point (i.e., Pre-1st iTBS, Post-1st iTBS, Pre-2nd iTBS, Post-2nd iTBS), 10 single-pulse stimuli were applied to the FDI region with a 5-second interval between pulses to ensure adequate MEP data collection.

To minimize potential carry-over effects, the three interval conditions (3, 5, and 7 hours) were tested on three separate experimental days for each subject, with a minimum washout period of 24 hours between sessions. The order of the intervals was randomized across subjects." These intervals are clinically feasible and sufficiently cover the effective time window between the two iTBS sessions. The MEP data were measured using peak-to-peak amplitude, and the average MEP amplitude for each time point was calculated. All data are presented as mean ± standard error (SEM).

Statistical Analysis

Repeated Measures ANOVA was used to assess differences between time points, and Bonferroni correction was applied for multiple comparisons.

Results

The MEP amplitude data recorded before and after the first and second iTBS sessions at 3 hours, 5 hours, and 7 hours showed that cortical excitability was continuously enhanced during the treatment interval. After the second stimulation, excitability remained elevated, indicating that the excitability effect from the first stimulation was not diminished. This provides physiological support for subsequent stimulation. The choice of a 3-7 hour interval is both clinically practical and aligned with experimental design requirements, further supporting the validity of the treatment interval.

| **eTable 1**. MEP Amplitude Changes at Different Time Points Following iTBS | | | | | |
| --- | --- | --- | --- | --- | --- |
| Interval | Time Point | Mean ± SD (mV) | Mean Difference | 95% CI | Adjusted P Value |
| 3 hours | Pre-1st iTBS | 0.4850 ± 0.067 | -0.6063 | -0.8505 to -0.3620 | < 0.0001 |
|  | Post-1st iTBS | 1.0913 ± 0.0920 |  |  |  |
|  | Pre-2nd iTBS | 0.8275 ± 0.1212 | -0.5113 | -0.7555 to -0.2670 | < 0.0001 |
|  | Post-2nd iTBS | 1.3388 ± 0.2829 |  |  |  |
| 5 hours | Pre-1st iTBS | 0.7488 ± 0.1290 | -0.4225 | -0.6667 to -0.1783 | 0.0001 |
|  | Post-1st iTBS | 1.1713 ± 0.1548 |  |  |  |
|  | Pre-2nd iTBS | 0.8913 ± 0.1850 | -0.3663 | -0.6105 to -0.1220 | 0.001 |
|  | Post-2nd iTBS | 1.2575 ± 0.0692 |  |  |  |
| 7 hours | Pre-1st iTBS | 0.6943 ± 0.1039 | -0.5825 | -0.8267 to -0.3383 | < 0.0001 |
|  | Post-1st iTBS | 1.2757 ± 0.2855 |  |  |  |
|  | Pre-2nd iTBS | 0.8214 ± 0.1385 | -0.7938 | -1.038 to -0.5495 | < 0.0001 |
|  | Post-2nd iTBS | 1.6243 ± 0.2813 |  |  |  |
| MEP: motor-evoked potentials. | | | | | |

| 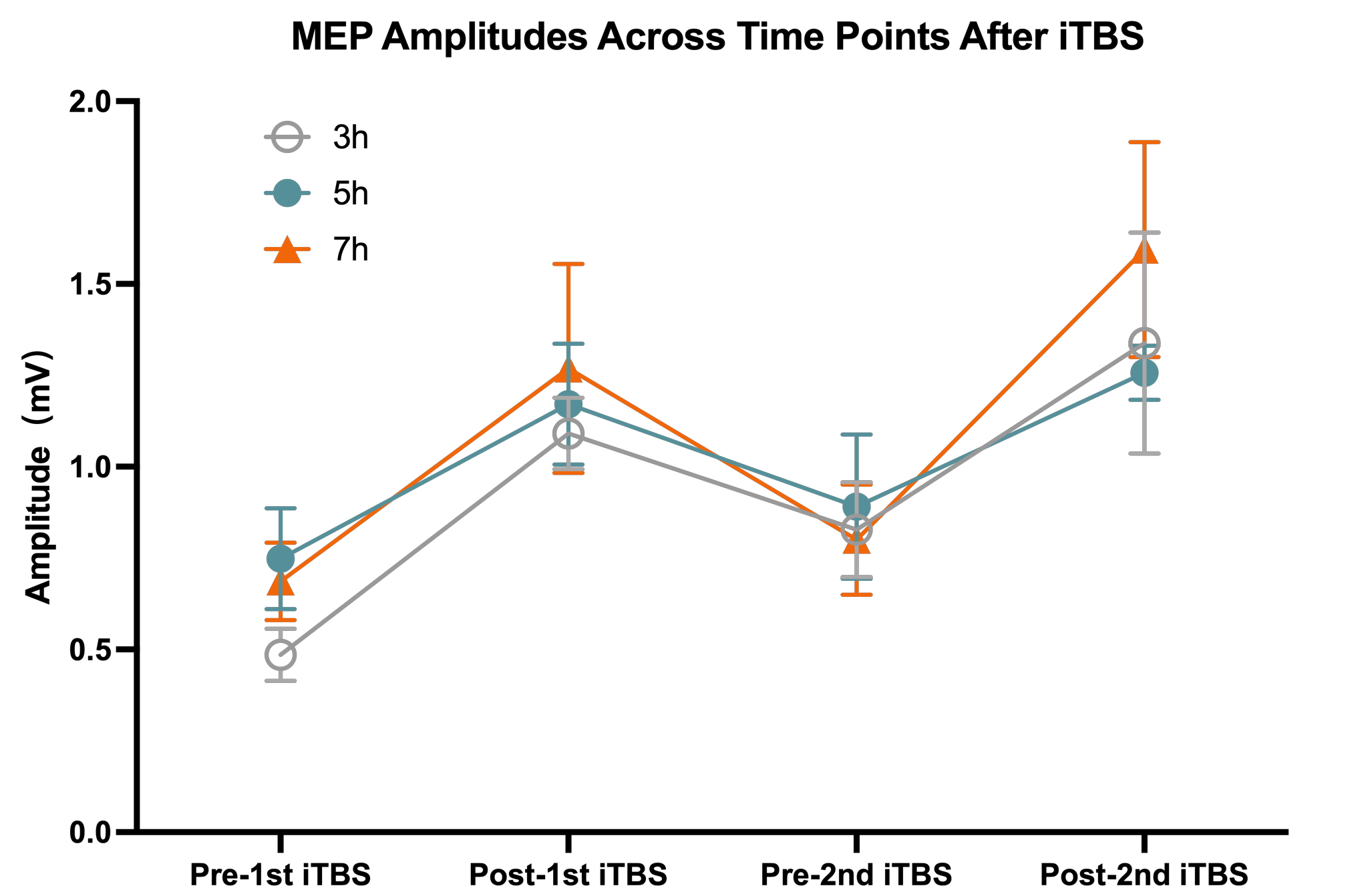 |
| --- |
| **eFig. 1.** MEP amplitudes measured before and after the first and second iTBS sessions at 3, 5, and 7 hours. Data are presented as mean ± SEM. MEPs were recorded from the contralateral first dorsal interosseous muscle, illustrating the temporal profile of cortical excitability across the inter-session interval. |
