# Supplementary material for: Intermittent theta-burst stimulation to enhance physical therapy in Parkinson's disease: The STEP-PD randomized trial
Source: Neurotherapeutics. 2026 Apr 1;23(3):e00897. doi: 10.1016/j.neurot.2026.e00897 (PMC13068805; doi:10.1016/j.neurot.2026.e00897)
Supplement: Multimedia component 2 [file mmc2.docx]

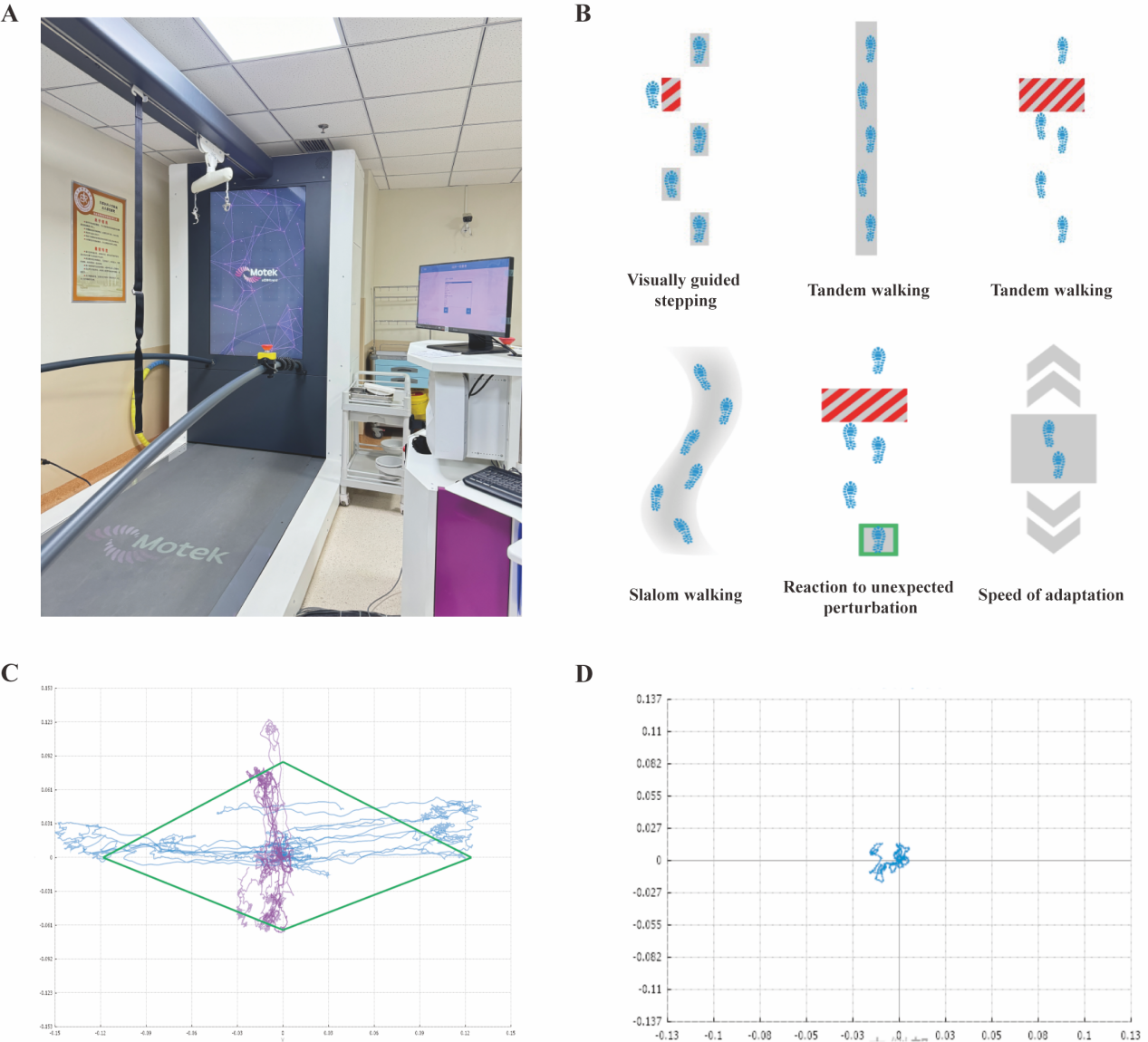


**eFig. 2.** The C-Mill gait device and its assessment system (Motek, Netherlands).

(A) The C-Mill located in the Parkinson’s Disease Assessment Room at the Neurorehabilitation Center of Beijing Rehabilitation Hospital. (B)The C-Gait task consists of six components; each component is scored on a scale from 0 to 100, representing task completion percentage, with higher scores indicating better performance: (1) Visually guided stepping, where the patient is required to step on projected targets that correspond to the current pattern; (2) Tandem walking, in which the patient must walk within the designated projection area; (3) Obstacle avoidance, where the patient needs to step over projected obstacles along the gait path; (4) Slalom walking, during which the patient must navigate a sinusoidal projection displayed on the treadmill; (5) Reaction to unexpected perturbations, where the patient responds to randomly appearing projected objects; and (6) Speed adaptation, where the patient walks within a designated square area while adjusting to changes in speed. (C) Limits of Stability: This evaluation quantifies the extent of stability in both the anterior-posterior and medio-lateral planes. The participant is instructed to stand with their feet aligned on the designated foot markers and to shift their upper body to the maximal extent possible in four specified directions (forward, backward, left, and right).(D) Postural Control: This evaluation assesses postural stability across four distinct tasks: standing with eyes open, standing with eyes closed, tandem stance, and unilateral stance. (E)The primary outcome measure is the displacement of the center of pressure (CoP) during these tasks, where smaller displacements indicate superior performance.
